# Supplementary material for: Antibody and cytokine levels in visceral leishmaniasis patients with varied parasitemia before, during, and after treatment in patients admitted to Arba Minch General Hospital, southern Ethiopia
Source: PLoS Negl Trop Dis. 2021 Aug 5;15(8):e0009632. doi: 10.1371/journal.pntd.0009632 (PMC8370634; doi:10.1371/journal.pntd.0009632)
Supplement: S1 Fig — (DOCX) [file pntd.0009632.s001.docx]

| **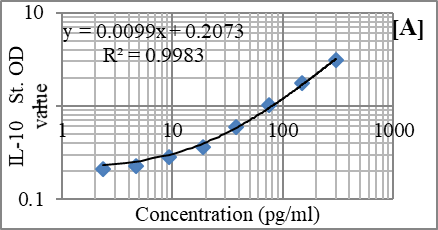** | **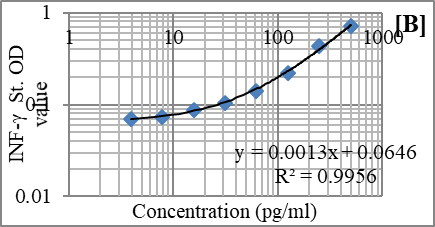** |
| --- | --- |
| **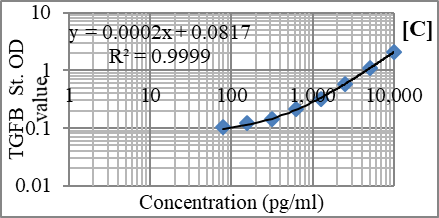** | **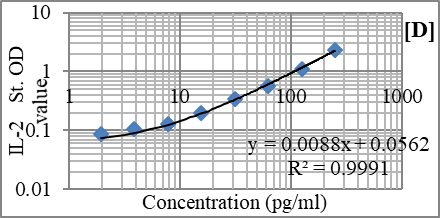** |

**S1 Fig: Standard curves of each cytokine concentrations.** A standard curve of each cytokine was obtained by measuring the OD values against the standard curve range concentration. **[A]** Standard curve of IL-10. **[B]** Standard curve of INF-γ. **[C]** Standard curve of TGF-β1. **[D]** Standard curve of IL-2 expressed as standards concentrations (pg/ml) per plate (96 reactions).
