# Supplementary material for: Antibody and cytokine levels in visceral leishmaniasis patients with varied parasitemia before, during, and after treatment in patients admitted to Arba Minch General Hospital, southern Ethiopia
Source: PLoS Negl Trop Dis. 2021 Aug 5;15(8):e0009632. doi: 10.1371/journal.pntd.0009632 (PMC8370634; doi:10.1371/journal.pntd.0009632)
Supplement: S2 Fig — (DOCX) [file pntd.0009632.s002.docx]

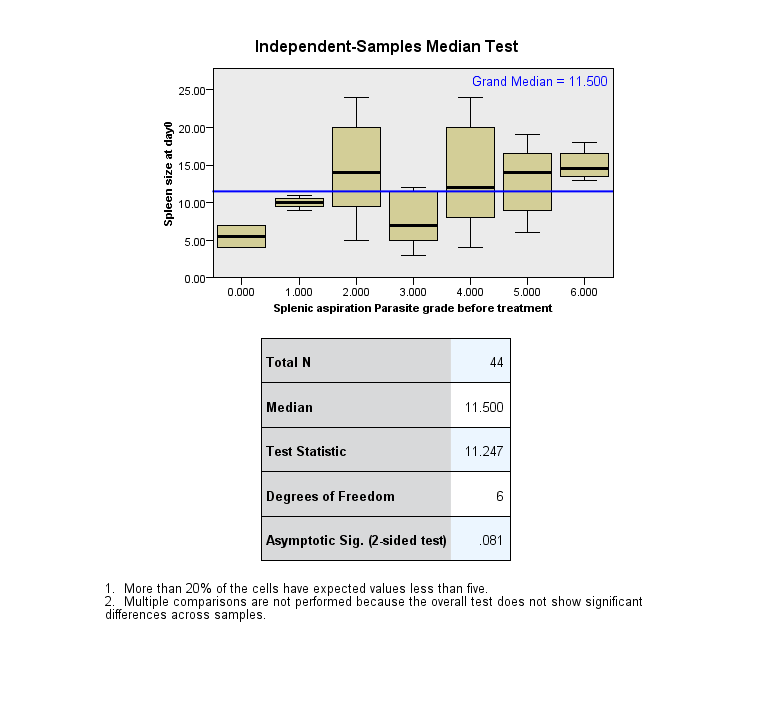

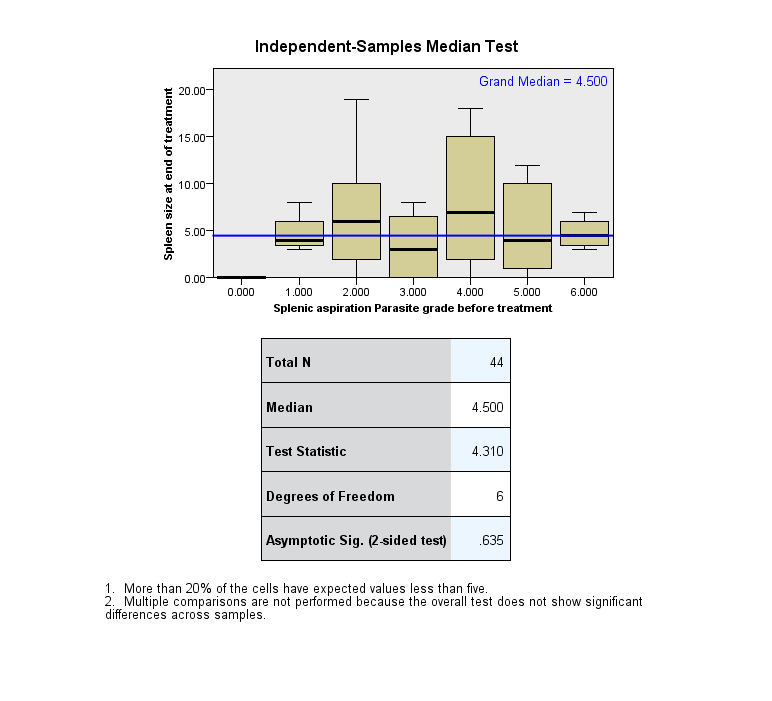


**[A]**

**[B]**

**[A]**

**S2 Fig:** **Box plots showing spleen size of VL patients at day 0 and end of treatment with varied grades of parasitemia (Independent sample median tests).** The y-axis shows spleen size in cm at **[A]** day 0. **[B]** EOT. The x-axis indicates splenic aspiration parasite grade with varied parasitemia as grade shown in S2 Table. Boxplots show the median (thick line across the box), interquartile range (vertical ends of the box), and whiskers (lines extending from the box to the highest and lowest values excluding the outliers and extremes). The horizontal reference line shows the grand median of spleen size (11.5 cm. at day 0 and 4.5 cm. at EOT).
