# Supplementary material for: Antibody and cytokine levels in visceral leishmaniasis patients with varied parasitemia before, during, and after treatment in patients admitted to Arba Minch General Hospital, southern Ethiopia
Source: PLoS Negl Trop Dis. 2021 Aug 5;15(8):e0009632. doi: 10.1371/journal.pntd.0009632 (PMC8370634; doi:10.1371/journal.pntd.0009632)
Supplement: S3 Fig — (DOCX) [file pntd.0009632.s003.docx]

**
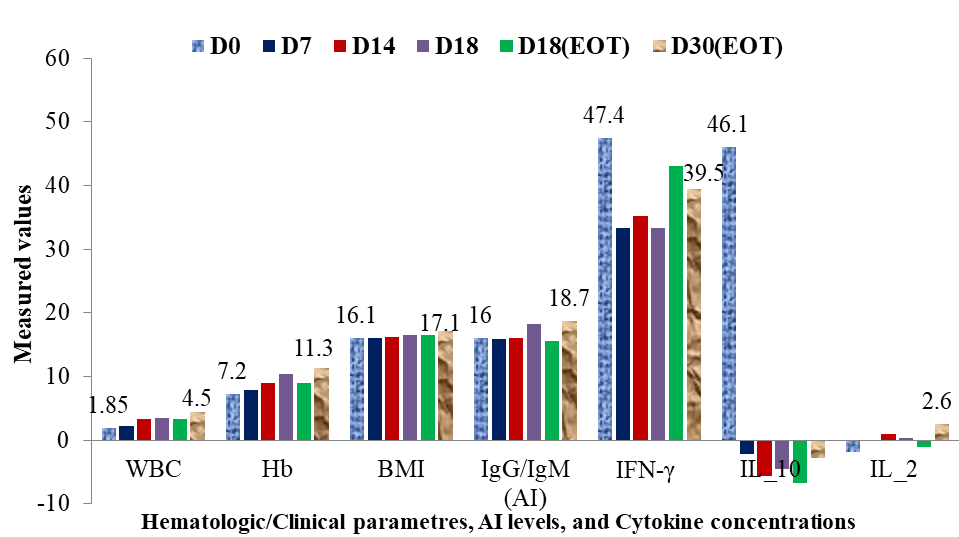
**

**S3 Fig: Comparison of hematologic and clinical parameters with levels of anti-leishmanial antibody and cytokine concentrations.** The x-axis in the bar graph shows grouped measured hematologic values of WBC and Hb, clinical values of BMI, levels of IgG/IgM (AI), and cytokine concentrations of IFN-γ, IL-10, and IL-2 meseared on active VL patients before treatment at base-line day 0 (n=48), during treatment at day 7 (n=48), day 14 (n=46), day 18 (n=11), and at day 18 EOT (n=36) for those who had been taken SSG + PM combination treatment and day 30 EOT (n=11) for those who had been taken SSG treatment alone. The y-axis shows median values WBC in x10^3^/mm^3^, Hb in g/dl, BMI in kg/m^2^, IgG/IgM (AI) ELISA O.D. values, and IFN-γ, IL-10, and IL-2 serum concentrations in pg/ml.
