# Supplementary material for: Antibody and cytokine levels in visceral leishmaniasis patients with varied parasitemia before, during, and after treatment in patients admitted to Arba Minch General Hospital, southern Ethiopia
Source: PLoS Negl Trop Dis. 2021 Aug 5;15(8):e0009632. doi: 10.1371/journal.pntd.0009632 (PMC8370634; doi:10.1371/journal.pntd.0009632)
Supplement: S1 Table — (DOCX) [file pntd.0009632.s004.docx]

**S1 Table:** Residence of study participants showing numbers of patients living in endemic area for VL and travel history

| **Region** | **Zone** | **Woreda** | **Kebele** | **Village** | **Travel History** | **# of VL cases** |
| --- | --- | --- | --- | --- | --- | --- |
| Afar | Afar (n=1) | Gewan | 01 |  | Omo | 1 |
| SNNPR | Amarokel (n=1) | Amaro | Yero | Sabafero | burjuji | 1 |
|  | Body (n=1) | Selemago | Gura | Shigdane |  | 1 |
|  | Daworo (n=2) | Lomma | Koyisha gorta | zama | Sudan | 1 |
|  |  | Lomma | Koyisha gorta | Ufo | Sudan | 1 |
|  | Gamo (n=1) | Bonke | Anekan | Achura | Uguma | 1 |
|  | Hadiya (n=1) | Gimbicho | Ombe Lange | Kareshancho | Metema | 1 |
|  | Hamar (n=5) | Hammer | Dimeka zuria | Yeka |  | 2 |
|  |  |  | Angude | Gadineba |  | 1 |
|  |  |  | Besheda | Gune |  | 1 |
|  |  |  | Zogona | gayana |  | 1 |
|  | Wolayita (n=2) | Sodo Zuria | Gurmowoyide | Maneara | Humera | 2 |
|  | Konso (n=28) | Konso | Abaroba | Galga |  | 2 |
|  |  |  |  | Orbaha |  | 1 |
|  |  |  |  | Sabafero |  | 1 |
|  |  |  |  | Kolmale |  | 1 |
|  |  |  |  | Amara |  | 2 |
|  |  |  |  | Gabo |  | 3 |
|  |  |  | Madaranagizaba | Hilte |  | 3 |
|  |  |  | Gelgelanakolmale | Boytate |  | 1 |
|  |  |  |  | Derogna |  | 1 |
|  |  |  |  | Halawge |  | 1 |
|  |  |  |  | Baytata |  | 1 |
|  |  |  |  | Ditata |  | 1 |
|  |  |  | Tsebelenakuchle | Sawale |  | 1 |
|  |  |  |  | Maruda |  | 1 |
|  |  |  |  | Kurata |  | 1 |
|  |  |  |  | Shane |  | 1 |
|  |  |  | Nalayasegen | Otaya |  | 1 |
|  |  |  |  | Kuyele |  | 1 |
|  |  |  | Masoya | Arkisha |  | 1 |
|  |  |  |  | Kette |  | 1 |
|  |  |  | Gera | Tetane |  | 1 |
|  |  |  | Sawgame | Modone |  | 1 |
| Oromia | Liben (n=3) | Liben | Alge | Soye |  | 1 |
|  |  |  | Hardot | Erbiba |  | 1 |
|  |  |  | Alge | Soye |  | 1 |
|  | Guji (n=3) | Gorodola | Dhuqo | Fulu |  | 1 |
|  |  |  | Raro | Tulajarti |  | 1 |
|  |  |  | Raro | Roka |  | 1 |
| **Total cases** | | | | | | **48** |
