# Supplementary material for: Antibody and cytokine levels in visceral leishmaniasis patients with varied parasitemia before, during, and after treatment in patients admitted to Arba Minch General Hospital, southern Ethiopia
Source: PLoS Negl Trop Dis. 2021 Aug 5;15(8):e0009632. doi: 10.1371/journal.pntd.0009632 (PMC8370634; doi:10.1371/journal.pntd.0009632)
Supplement: S2 Table — (DOCX) [file pntd.0009632.s005.docx]

**S2 Table: Parasite grading procedures in smears od splenic tissue:** parasite load grades are derived from logarithmic transformations of parasite counts re-classified as ordinal data from 0 to 6, where 0 grade is no parasites per 1000 microscopic fields (1000x), grade 1 is 1–10 parasites per 1000 fields, grade 2 is 11–100 parasite per 1000 fields, grade 3 is 101–1,000 parasites per 1000 fields, grade 4 is 1001–10,000 parasites per 1000 fields, grade 5 is 10,001–100,000 parasites per 1000 fields and, grade 6 is greater than 100,000 parasites per 1000 fields (>100 parasites per field) .

| Number of amastigotes per 1000 fields | Grade |
| --- | --- |
| Greater than 100,000 | 6 |
| 10,001 – 100,000 | 5 |
| 1,001 – 10,000 | 4 |
| 101 – 1,000 | 3 |
| 11 – 100 | 2 |
| 1 – 10 | 1 |
| 0 | 0 |
