# Supplementary material for: Antibody and cytokine levels in visceral leishmaniasis patients with varied parasitemia before, during, and after treatment in patients admitted to Arba Minch General Hospital, southern Ethiopia
Source: PLoS Negl Trop Dis. 2021 Aug 5;15(8):e0009632. doi: 10.1371/journal.pntd.0009632 (PMC8370634; doi:10.1371/journal.pntd.0009632)
Supplement: S3 Table — (DOCX) [file pntd.0009632.s006.docx]

**S3 Table.** Description of the study population by socio-demographic characteristics, and clinical/laboratory parameters at base line and EOT (Median and IQ Range)

| Type of variables | N (%) | Median | Interquartile (IQ) Range |
| --- | --- | --- | --- |
| Sex Male | 38 (79.2) |  |  |
| Female | 1. (20.8) |  |  |
| Age in years | 48 | 18.50 | 10.00−25.00  (Min. 5.00 − Max. 58.00) |
| Occupation Farmer  Student  Other | 33 (68)  2 (4.2)  13 (27.1) |  |  |
| VL endemic area Yes  No | 39 (81.3)   1. (18.8) |  |  |
| Fever at admission Yes | 1. (100) |  |  |
| k39 test Positive | 1. (100) |  |  |
| HIV test Negative | 48 (100) |  |  |
| VL treatment PM + SSG  SSG alone | 35 (76)   1. (24) |  |  |
| Duration of illness in months (n=48) | - | 3.00 | 2.00−4.25 |
| BMI in kg/m^2^  Day 0 (n=48) | - | 15.80 | 14.30−18.00 |
| EOT (n=46) | - | 16.20 | 14.80−18.40 |
| Spleen size in cm Day 0 (n=48) | - | 11.00 | 7.25−15.75 |
| EOT (n=46) | - | 5.00 | 1.5−8.00 |
| Hemoglobin in (g/dl) Day 0 (n=48) | - | 7.25 | 6.35−8.63 |
| EOT (n=46) | - | 9.35 | 8.65−10.45 |
| RBC (x10^6^/µL) Day 0 (n=48) | - | 3.12 | 2.86−3.51 |
| EOT (n=46) | - | 3.87 | 3.46−4.39 |
| WBC (x10^3^/mm^3^) Day 0 (n=48) | - | 1.90 | 1.4−3.00 |
| EOT (n=46) | - | 3.55 | 2.7−4.53 |
| Platelet (x10^3^/mm^3^) Day 0 (n=48) | - | 98.00 | 65.25−153.00 |
| EOT (n=46) | - | 260.00 | 174.25−307.50 |
