# Supplementary material for: Antibody and cytokine levels in visceral leishmaniasis patients with varied parasitemia before, during, and after treatment in patients admitted to Arba Minch General Hospital, southern Ethiopia
Source: PLoS Negl Trop Dis. 2021 Aug 5;15(8):e0009632. doi: 10.1371/journal.pntd.0009632 (PMC8370634; doi:10.1371/journal.pntd.0009632)
Supplement: S4 Table — (DOCX) [file pntd.0009632.s007.docx]

**S4 Table:** Comparison of the study population socio-demographic characteristics, and clinical/laboratory parameters with age group at base line (Median and IQ Range)

| Age of VL patients | Age 5 to 17 (n=23) | | Age >17 (n=25) | |
| --- | --- | --- | --- | --- |
|  | Median (%) | IQ range | Median (%) | IQ Range |
| Sex Male | 16 (72.7) |  | 22 (84.6) |  |
| Female | 6 (22.3) |  | 4 (15.4) |  |
| VL endemic area Yes  No | 21 (81.3)  1 (18.8) |  | 18 (62.2)  8 (30.8) |  |
| VL treatment PM + SSG  SSG alone | 16 (72.7)  6 (22.3) |  | 19 (79.2)  5 (20.8) |  |
| Duration of illness in months (n=48) | 2−3 | 2 | 2 | 2−3 |
| BMI in kg/m^2^  Day 0 (n=48) | 14.3 | 13.4−14.8 | 17.3 | 16.2−18.2 |
| Spleen size in cm Day 0 (n=48) | 13.5 | 10−17 | 12 | 6−16 |
| EOT (n=46) | 5.5 | 0−6 | 0 | 0 |
| Hemoglobin in (g/dl) Day 0 (n=48) | 6.9 | 5.8−8.4 | 7.4 | 6.5−8.4 |
| EOT (n=46) | 9.65 | 8.7−10.3 | 8.95 | 8.5−10.65 |
| RBC (x10^6^/µL) Day 0 (n=48) | 3.14 | 2.85−3.82 | 3.12 | 2.84−3.38 |
| EOT (n=46) | 3.96 | 3.56−4.87 | 3.78 | 3.32−4.29 |
| WBC (x10^3^/mm^3^) Day 0 (n=48) | 2.1 | 1.5−3.4 | 1.7 | 1.2−2.2 |
| EOT (n=46) | 4.05 | 3.5−5.7 | 5.6 | 3.3−6.4 |
| Platelet (x10^3^/mm^3^) Day 0 (n=48) | 126 | 50−175 | 148 | 126−268 |
| EOT (n=46) | 274 | 211−294 | 246 | 230−287 |
| IgG/IgM (AI) at Day 0 | 16.97 | 15.46−19.58 | 15.83 | 13.62−19.03 |
| INF-γ (pg/ml) at Day 0 | 36.74 | 23.28−161.08 | 55.4 | 18.83−139.94 |
| TGFβ1 (pg/ml) at Day 0 | 6490 | 3265−9665 | 5715 | 4565−8015 |
| IL_10 (pg/ml) at Day 0 | 59.05 | 26.57−84.40 | 48.45 | 17.7−59.9 |
| IL_2 (pg/ml) at Day 0 | -1.35 | -3−4.98 | -2.82 | -4.26−-3 |
