# Supplementary material for: Antibody and cytokine levels in visceral leishmaniasis patients with varied parasitemia before, during, and after treatment in patients admitted to Arba Minch General Hospital, southern Ethiopia
Source: PLoS Negl Trop Dis. 2021 Aug 5;15(8):e0009632. doi: 10.1371/journal.pntd.0009632 (PMC8370634; doi:10.1371/journal.pntd.0009632)
Supplement: S5 Table — (DOCX) [file pntd.0009632.s008.docx]

**S5 Table:** Comparison of the study population socio-demographic characteristics, and clinical/laboratory parameters with treatment type at base line (Median and IQ Range)

| Age of VL patients | PM + SSG (n=35) | | SSG alone (n=11) | |
| --- | --- | --- | --- | --- |
|  | Median (%) | IQ range | Median (%) | IQ Range |
| BMI in kg/m^2^  Day 0 (n=48) | 15.9 | 14.2−17.8 | 57.7 | 14.8−17.3 |
| Spleen size in cm Day 0 (n=48) | 14 | 11−17.5 | 6 | 4−10 |
| EOT (n=46) | 7 | 3.5−10 | 0 | 0−5 |
| Hemoglobin in (g/dl) Day 0 (n=48) | 6.8 | 5.3−7.8 | 8.4 | 7.0−9.5 |
| EOT (n=46) | 8.9 | 8.25−10.2 | 10.9 | 10.0−11.85 |
| RBC (x10^6^/µL) Day 0 (n=48) | 3.04 | 2.76−3.38 | 3.61 | 2.11−3.93 |
| EOT (n=46) | 3.68 | 3.36−4.22 | 4.52 | 4.15−4.76 |
| WBC (x10^3^/mm^3^) Day 0 (n=48) | 1.8 | 1.3−2.5 | 2.0 | 1.5−3.0 |
| EOT (n=46) | 3.3 | 2.6−3.95 | 3.6 | 3.3−5.7 |
| Platelet (x10^3^/mm^3^) Day 0 (n=48) | 94 | 58−126 | 126 | 91−162 |
| EOT (n=46) | 239 | 163−303 | 287 | 246−294 |
| IgG/IgM (AI) at Day 0 | 15.77 | 13.82−18.62 | 19.29 | 16.31−24.93 |
| INF-γ (pg/ml) at Day 0 | 36.33 | 20.36−117.21 | 129.32 | 51.42−162.62 |
| TGFβ1 (pg/ml) at Day 0 | 5715 | 3665−8365 | 7265 | 4565−7665 |
| IL_10 (pg/ml) at Day 0 | 58.10 | 25.34−79.89 | 31.43 | 15.24−50.8 |
| IL_2 (pg/ml) at Day 0 | -1.62 | -3.31−2.26 | -2.49 | -3.46—1.61 |
