# Supplementary material for: Antibody and cytokine levels in visceral leishmaniasis patients with varied parasitemia before, during, and after treatment in patients admitted to Arba Minch General Hospital, southern Ethiopia
Source: PLoS Negl Trop Dis. 2021 Aug 5;15(8):e0009632. doi: 10.1371/journal.pntd.0009632 (PMC8370634; doi:10.1371/journal.pntd.0009632)
Supplement: S6 Table — (DOCX) [file pntd.0009632.s009.docx]

**S6 Table: Mean/median AI values of IgG/IgM and cytokine concentrations** in sera of active VL patients collected before treatment, at various follow-up schedules post-treatment, and at end of treatment (day18/30).

| Measurements | N | Mean | Median (IQ range) |
| --- | --- | --- | --- |
| IgG/IgM (AI) D0 | 48 | 16.4 | 16.0 (13.6−19.3) |
| IgG/IgM (AI) D7 | 46 | 16.7 | 15.9 (13.5−18.1) |
| IgG/IgM (AI) D14 | 46 | 16.4 | 16.0 (14.1−19.9) |
| IgG/IgM (AI) D18 | 11 | 19.3 | 18.3 (17.6−23.9) |
| IgG/IgM (AI) D18/EOT | 36 | 15.1 | 15.5 (11.5−17.4) |
| IgG/IgM (AI) D30/EOT | 11 | 17.7 | 18.7 (16.9−19.6) |
| IgG/IgM (AI) D120 | 26 | 9.8 | 9.6 (6.8−12.3) |
| IgG/IgM (AI) HC | 20 | 4.5 | 4.6 (3.4−5.7) |
| IFN-γ (pg/ml) D0 | 48 | 106.1 | 47.4 (21.9−134.6) |
| IFN-γ (pg/ml) D7 | 46 | 80.6 | 33.3 (19.7−68.5) |
| IFN-γ (pg/ml) D14 | 46 | 64.6 | 35.2 (16.0−65.9) |
| IFN-γ (pg/ml) D18 | 11 | 45.3 | 33.4 (26.2−56.5) |
| IFN-γ (pg/ml) D18/EOT | 36 | 85.1 | 43.0 (19.3−80.3) |
| IFN-γ (pg/ml) D30/EOT | 11 | 47.7 | 39.5 (28.8−78.3) |
| IFN-γ (pg/ml) D120 | 26 | 56.8 | 33.5 (13.3−74.1) |
| INF-y (pg/ml) HC | 20 | 8.1 | 8.0 (2.6−10.7) |
| TGFβ1 (pg/ml) D0 | 48 | 6739.5 | 6215.0 (4090.0−8690.0) |
| TGFβ1 (pg/ml) D7 | 46 | 8092.2 | 8390.0 (4815.0−10315.0) |
| TGFβ1 (pg/ml) D14 | 46 | 8202.0 | 7540.0 (4740.0−10465.0) |
| TGFβ1 (pg/ml) D18 | 11 | 8496.8 | 8515.0 (7165.0−10365.0) |
| TGFβ1 (pg/ml) D18/EOT | 36 | 8891.4 | 8340.0 (5927.5−13040.0) |
| TGFβ1 (pg/ml) D30/EOT | 11 | 9219.5 | 8365.0 (7265.0−11665.0) |
| TGFβ1 (pg/ml) D120 | 26 | 8169.0 | 6365.0 (4290.0−10540.0) |
| TGFβ1 (pg/ml) HC | 20 | 5540.0 | 5990.0 (3752.5−6840.0) |
| IL_10 (pg/ml) D0 | 48 | 47.9 | 46.1 (16.5−71.7) |
| IL_10 (pg/ml) D7 | 46 | -1.6 | -2.1 (-13.5−6.1) |
| IL_10 (pg/ml) D14 | 46 | -5.4 | -5.6 (-19.8−-1.6) |
| IL_10 (pg/ml) D18 | 11 | -4.8 | -4.5 (-7.6−0.3) |
| IL_10 (pg/ml) D18/EOT | 36 | -9.2 | -6.8 (-20.0−-2.2) |
| IL_10 (pg/ml) D31/EOT | 11 | -2.6 | -2.8 (-6.9−-1.6) |
| IL_10 (pg/ml) D120 | 26 | -11.3 | -8.5 (-20.7−-7.1) |
| IL-10 (pg/ml) HC | 20 | -20.5 | -20.5 (-22.6−-19.6) |
| IL_2 (pg/ml) D0 | 48 | 2.2 | -1.8 (-3.4−3.2) |
| IL_2 (pg/ml) D 7 | 46 | 3.6 | -0.1 (-2.8−7.1) |
| IL_2 (pg/ml) D14 | 46 | 6.0 | 1.0 (-2.1−8.4) |
| IL_2 (pg/ml) D18 | 11 | 8.6 | 0.3 (-2.0−24.0) |
| IL_2 (pg/ml) D18/EOT | 34 | 0.6 | -1.1 (-3.41.9) |
| IL_2 (pg/ml) D30/EOT | 11 | 4.8 | 2.6 (-1.9−8.1) |
| IL_2 (pg/ml) D120 | 26 | 2.5 | 0.6 (-2.2−3.9) |
| IL-2 (pg/ml) HC | 20 | 5.5 | 3.5 (1.2−9.3) |
