# Supplementary material for: Antibody and cytokine levels in visceral leishmaniasis patients with varied parasitemia before, during, and after treatment in patients admitted to Arba Minch General Hospital, southern Ethiopia
Source: PLoS Negl Trop Dis. 2021 Aug 5;15(8):e0009632. doi: 10.1371/journal.pntd.0009632 (PMC8370634; doi:10.1371/journal.pntd.0009632)
Supplement: S1 Procedure — (DOCX) [file pntd.0009632.s010.docx]

**S1 Procedure: Indirect ELISA for detection of anti-leishmania antibody**

Briefly, 100 µL of serum dilution solutions were added to all wells of microtiter plate. Then, 5 µL of each samples, 5 µL of positive control, 5 µL of cutoff serum (in duplicates) and 5 µL of negative control were added into the corresponding wells and incubated at 37±1 ºC for 45 min. After extensive wash with EL_X_ 800 Bioelisa Washer (Biokit, Highland Park, USA) to remove unbound antibodies, 100 µL of conjugate solutions were added into each well, sealed and incubated at 37±1 ºC for 30 min. After further washing, 100 µL/well of substrate solution Tetramethylbenzidine (TMB) was added and incubated at room temperature for 20 min. After adding 50 µL of stopping solution in each well, the absorbance of the antigen-antibody-conjugate-substrate solution was read with EL_X_ 800 Bioelisa Reader (Biokit, Highland Park, USA) at 450/630 nm.
