# Supplementary material for: Antibody and cytokine levels in visceral leishmaniasis patients with varied parasitemia before, during, and after treatment in patients admitted to Arba Minch General Hospital, southern Ethiopia
Source: PLoS Negl Trop Dis. 2021 Aug 5;15(8):e0009632. doi: 10.1371/journal.pntd.0009632 (PMC8370634; doi:10.1371/journal.pntd.0009632)
Supplement: S2 Procedure — (DOCX) [file pntd.0009632.s011.docx]

**S2 Procedure: ELISA procedure for measuring cytokine concentrations (IL-10, IFN-γ, TGF-β1, and IL-2)**

Briefly, 100 μL/well of capture antibody was coated in 96-well microtiter plate (Corning Costar 9018, or Nunc Maxisorp®) with specific capture antibodies against one of the four cytokines (IFN-γ, TGFβ1, IL-10, and IL-2). Plates were incubated overnight at 2-8 ºC. Standards and control samples were included in each plate along with diluted patient’s serum samples. After 2 hours of incubation, followed by extensive wash, 100 μL/well of biotinylated detection antibodies was added. Finally, 100 μL/well of TMB was added and incubated for 15 minutes. Light absorbance at 450/630 nm of the streptavidin-HRP and TMB complex was measured with ELISA Reader.
