# Supplementary material for: Antibody and cytokine levels in visceral leishmaniasis patients with varied parasitemia before, during, and after treatment in patients admitted to Arba Minch General Hospital, southern Ethiopia
Source: PLoS Negl Trop Dis. 2021 Aug 5;15(8):e0009632. doi: 10.1371/journal.pntd.0009632 (PMC8370634; doi:10.1371/journal.pntd.0009632)
Supplement: Attachment — Submitted filename: Responses to the Reviewers comments & Questions.docx [file pntd.0009632.s013.docx]

| **Responses to the Reviewers' comments**  First of all, we are very much thankful for the comments and suggestions of reviewers which had substantially improved the quality of the manuscript. Reviewers’ comments were considered carefully for revising the manuscript. | |
| --- | --- |
| **Editors comment** | **Responses to the Reviewers' comments** |
| **Reviewer #1:** |  |
| Analyzes of cytokines such as IL-12 and TNF-alpha could be included in this study. | Due to shortage of resources, we are unable to perform these experiments. But we will address this in our future studies. |
| Future analyzes should not be included in the conclusion. | Future analysis are removed from the conclusion part in the revised manuscript |
| lines 34-35: AI levels decrease in healthy controls? | Healthy controls were tested once for comparison purpose and a lower value of AI was measured. There were no repeated measurements, and no follow-up was planned. Thus, corrections were made to reflect this. |
| line 37: clinical-epidemiological | Corrected as suggested in the revised manuscript. |
| Introduction: better describe the role of cytokines related to this study (IL-2, TGF-beta and IFN-gamma). | Role of all cytokines are elaborated in the revised manuscript, (line # 75 – 96) |
| line 200: Fig 1 could be shown in the supporting information. | Fig 1 is shown in the supporting information in the revised manuscript as S1 Fig |
| line 241: change to 76% | Percentage was corrected in the revised manuscript. |
| Table 2: review the "variables" column (EOT) | Corrected in the revised manuscript. |
| Reviewer #2: |  |
| 1- Our approach is to understand how the immune response generated during different treatment stages of active VL before, during and post-treatment that are impacted by the presence of different cytokines resulting in clinical pathogenesis and disease. This sentence could be better if replaced by the one below  Our approach is to understand how the immune response generated during different treatment stages of active VL before, during, and post-treatment is impacted by the presence of different cytokines resulting in clinical pathogenesis and disease. | Rephrased and revised in the revised manuscript. |
| 2- …. found in areas of L. donovani transmission >>>> found in *L. donovani* transmission areas … | Corrected |
| 3- In Ethiopia, with an estimated 4,500 to 5,000 new cases occur every year, the population at risk is more than 3.2 million  >>>Could be reformulated with an easier way | Rephrased in the revised manuscript |
| 4- in the Eastern Africa Region, new challenges of VL control attributed to lack of appropriate tools and HIV co-infection have eventually led to resurgence of leishmaniasis and its spread to new geographical areas.  >>> what is the meaning of “lake of appropriate tools” ? Please clarify this sentence or remove it. (The introduction could be shortened by keeping only the essential information in consistence with the research question) | We are sorry about the editorial problem. The word should read as “lack” instead of “lake”.  Rephrased, corrected and revised. |
| 5- Untreated VL patients act as reservoirs for parasites and therefore contribute to disease transmission in anthroponotic VL areas  >>>> please provide a good reference that explain clearly the anthroponotic VL transmission (Directly from human to human by only a phlebotomy or another vector) in Ethiopia or elsewhere. | Relevant references have been included in the revised manuscript, (Line # 56 – 61) |
| 6- In the introduction section, the authors explain the usefulness of a test of VL cure in the context of the HIV coinfected patients. However, the 48 patients selected for the study did not have any detailed information about their HIV condition. Please provide the results based on this criterion. Or focus of the manuscript on the immunocompetent patients. The only information is about POSITIVITY OF THE TEST but not the immunodeficiency state which interact with the antibody levels | Since there were no HIV positive patients, sentences pertaining to the HIV status of patients were removed from the revised manuscript. |
| 7- Just the next paragraph, the authors explain the importance of asymptomatic VL persons to be detected>>> same remark for the study. Only Active VL was selected. | In the present study there were no asymptomatic VL patients and therefore we removed the sentence describing the same in the revised manuscript. |
| 8- The paragraphs that include the presentation of markers and their link to VL and the immunity need a better reformulation by focusing on the main questions of this study. (What could be remembered is that the authors are formulating questions about the role of VL treatment and they presented only the increase of markers in case of active VL what about the asymptomatic and the HIV infected persons, what about the provided treatment …etc) | The research was conducted on active VL patients’ antibody levels and cytokine concentrations at different time point during treatment periods. Asymptomatic VL, HIV-VL coinfected patients and treatment efficacy are beyond the scope of the present research work. Any text related to asymptomatic and HIV co-infection has been modified or deleted in the revised manuscript. |
| 9- “Concerning prognostic tools, it is a worthful effort testing if concentrations of selected cytokines in serum could be used to monitor treatment outcomes.”  >> it could be better to start with this reflexion and find relevant references to explain which kind of cytokines. | The sentence was rephrased and relevant references were cited in the revised manuscript, (Line # 74) |
| 10- The study is a clinical laboratory study that needs to be presented based on an appropriate guideline and checklist (Please find the suitable one in the EQUATOR network website). | Guidelines and checklist for this clinical study was referred and utilized from the EQUATOR network. |
| 11-    The results could be compared to the accuracy of the rk39 rapid test used. Is it one rapid diagnostic test (As cited InBios*) or different kind of trademark? In case of many trademark what are the known accuracy and how the authors re-confirmed the diagnostic. | In this study only one brand of rk39 rapid test was used, that is InBios International, Inc. Seattle, WA, USA. (Line # 137 & 219 – 220) |
| 12-    This is really “A prospective cohort study” Definitely not. What is described is the way to get the blood samples to start the laboratory study. (May be the authors intend to present another manuscript putting on the spot such prospective cohort study) But for the actual manuscript focusing on the laboratory results is the best strategy. | The study design was corrected as a descriptive cross-sectional study, but incorporating follow-up sampling 120 after treatment. |
| 13-    If already the study involving the recruited patients as cited “ A prospective cohort study was conducted among admitted VL patients who were treated and followed at different time points between 02/September/2015 to 01/August/2016” >>> Please provide the reference and focus on the origin of the blood samples. | This research was not related or linked to another study. The study report is based on a stand-alone descriptive cross-sectional design. |
| 14-    All this information should be shown as a supplementary material and presented as table and combine in excel form all the information provided in S1 table. There is a need to show who take the combination or the monotherapy (combination treatment of Paromomycin Sulphate (PM) and Sodium Stibogluconate (SSG) or day 30 for those who treated with Sodium Stibogluconate (SSG) monotherapy) The results normally will be presented based on this difference of received treatment. | The tables are substantially revised and included as supplementary files. During the study period, 11 of the 48 patients were treated with SSG alone for 30 days, since paromomycin was not available. The number of patients treated with 30 days is small, and the study was not powered to analyse the data by treatment regimens administered. However, similar observation is noted when data is analyzed by the type of treatment taken in (S5 Table). |
| 15-    There is a need of precising HIV conditions of the patients? If the information is available to be consistent with the reflexion in the introduction section. | During our study period, all study participants were tested negative for HIV. The introduction part was corrected with HIV negative VL patients in the revised manuscript. |
| 16-    For this study, it’s important to precise how the samples were managed stored in which conditions and if there is any messing data with regard the no possibility to get all blood sampling in the targeted days. | Samples were stored at -20 ^0^C and -80^0^C in the deep freezer. We have included the data of patients with adequate follow-up. Some patient sera during the study were not available, for instance day 0 (n=1) and after 3^rd^ month follow up at day 120 (n=20). Details are included in the revised manuscript. (Line # 153 – 157) |
| 17-    Since 1990s, ample evidence has been generated that a combination of the WHO clinical case definition for VL and a positive antibody test is an adequate for decision to treat [22,23]. >>>> There is no need for such explanation. Or you can move it to an appropriate place in the introduction section. | Removed from the revised manuscript |
| 18-    How many technicians or may be the authors involved to count and put the index for microscopy. The graded scale was used with only one and same person; what is the degree of coherence between two persons who did this grading. Please add kappa coefficient and explain if any pictures of the amastigots L.donovani forms were taken and how the slides were stored.  Could you add pictures for each of the 6 grades as Table (Figure)S2 | Two technicians counted parasites in 1000 microscopic fields under oil immersion (1000x magnification). No two technicians will read similar microscopic fields, and thus variability in absolute counts is expected. However, the grading system and the assignment of grades accommodate a one log difference of counts by two microscopists. For instance, counts of 99 and 999 parasites respectively will have grades of 2 and 3. Such a difference in counting that leads to more than one log is a rare event. Mostly, counts with a one log difference will have a similar grade. For instance, counts of 101 and 999 parasites per 1000 fields will both have a parasite grade of 3. Often a difference of 1 log is the worst scenario, which can be of importance when grades are either assigned at 0 or 1, whereby a parasite grade of 0 leads to false negative results during diagnosis of VL. This did not happen in our case, since all the study participants have a parasitological confirmation of VL. Due to this, and because the grading system is inherently variable, it would not make sense to determine kappa values. Further, we did not plan to take pictures of smears as this was a very routine practise in our clinic. |
| 19-    To shorten the Method section please add the full technic to analyse Indirect ELISA for detection of anti-leishmania antibody and the Cytokine Profile (IL-10, IFN-γ, TGF-β1, and IL-2) as a supplementary material and keep just some words in the method section. | Data were included in the supplementary files in S1 & S2 Procedure; S1 Fig. |
| 20-    For the ethical clearance, if the study is already published elsewhere, is important to mention if the authors where allowed to take the stored blood samples for doing this study | Not published elsewhere, and blood sample were collected directly from the participants for the purpose of this study only. |
| 21-    As the legal age is 18 y o Please provide the age range between 5 and 17yo in replacement of the sentence (the age ranges of 5-58. Higher proportions (79.2%)) | The necessary corrections concerning the figure 79.2% has been made. The median age of the study participants is 18.5 (S3 Table). An additional table is provided to show age distribution in children and adults, also shown by gender (S4 Table). We hope that gives a more transparent data concerning age distribution. |
| 22-    If the children represent almost the half of the blood samples, please explain in the discussion part if the biomarkers targeted had different concentrations based on the age group of the normal population without including the VL condition. Just to be sure that the potential confounding factors are neutralized. | Indeed, there were 23 children (<17 yo) from the total 48 study participants. However, majority of them were in fact adolescents. In this study, a normal population from an endemic has not been included. The purpose was to compare cytokine concentrations and antibody index values in confirmed VL patients with those of unexposed controls, we have sampled blood donors representing healthy donors, which were also 18 years and above. Due to this, we are not in a position to address this comment of the respected reviewer. |
| 23-    What is the reason of treating some patient with a combined ttt and the others less than 24% by a monotherapy? All patients were hospitalized by the duration of the hospitalisation is not noticed (please in the Future Excel file it could be very informative to add the ages and the treatment provided, doses and duration of the hospitalisation, with the concentration levels of the laboratories parameters for each of the 48 blood samples analysed. | The study did not put in place a specific treatment regimen. All patients were to be treated using the national guideline, i.e. using a combination of SSG and PM for 17 days, as per the WHO recommendations. Unfortunately, PM was not available for some patients, and thus 11 patients had to be treated with SSG monotherapy for 30 days. Explanation why patient received different treatment regimens is now included in the revised manuscript. Further, we have availed detailed information (duration of illness, treatment doses and durations, etc.) for each patient in a Supplementary excel file. All patients were treated hospitalized. |
| 24-    TABLE 1 does not give any important information by seeing Median and interquartile statistics to help understanding the correlation between the socio demographic characteristics and the level of parasitemia or the level of Interferon gamma or the cytokines. A better information could be provided with a p value to show which socio demographic characteristics could or not influence the laboratory parameters or Microscopy grade parasetemia in each targeted date (D0, D7 D18 …D120) (The small sample will be managed with fisher exact analysis) | In addressing the important comment of the reviewer, Table 1 has been moved to the supplementary materials. The data on cytokines and AI values are summarized in figures 4, 5 & 6, which show trends with treatment duration (from day 0 to 7, 14, 18, 30 and 120 days) in patients with varied parasitaemia. Nonetheless table 1 which is now S3 table gives the descriptive characteristics of the patients, and it was not intended to show the correlation between socio-demographic characteristics (age, sex, etc.) and level of parasitaemia and cytokines.  A sub-group analysis of data on parasitaemia, cytokines and AI values by age groups and gender did not reveal significant differences from data analysed globally; that is, the trends remained the same. This data is not shown in tables and figures but mentioned in S4 Table. The manuscript is already replete with many figures, which we have to refrain to add more. |
| 25-      Same remark for all the rest of the presented information in the result section. Too many that need to be synthetised with remind that in general for one manuscript 5 tables or Figures should be on the main text to be attractive and easy to read.   - The main problem in the presentation of the results is the absence of the standardised comparison. Based on the introduction section, the main objective is to show active vs asymptomatic cases (is not possible from the collected data) the other main objective is to present HIV positive and HIV negative cases and the influence of such condition on the results. Another main objective is the before and after treatment results. The other main information is based on the potential Cytokines concentration to decrease with the cure state. - One of the propositions to readdress the heavy information in the result section is to focus primarily on the cure test, which is based on the initial bone marrow parasitological grade in Microscopy and the final bone marrow parasitological grade in the microscopy (after getting all the treatment) The treatment effectiveness is the first information that could be analysed. - Then the comparison on the laboratory markers change before starting the treatment during the treatment and after the treatment become interesting to provide in very concise way. - Secondly, on the concentration change of the cytokines compared to the different groups of comparison ttt, hiv condition, sympt vs asympto, and the main socio demographic characteristics (which is not possible as all the 48 cases are only active VL cases, and the HIV condition is not available) | Some figures and tables have been moved to supplementary material  Since our study exclusively focused on cytokines profiles, antibody levels and other clinical and laboratory parameters at various time points and the relationship to the treatment outcome among active VL patients, sentences related to HIV positive patients and asymptomatic VL patients were deleted from the introduction section. The comparisons are with healthy controls (blood donors) and before, during and after completion of treatment. We also considered comparisons with a 120-day follow-up sampling.  The efficacy of the treatment is not included in the present study. However, all patients except one were successfully treated. The exceptional patient was treated with second-line treatment and ultimately cured. This was described in the original submission and appears similarly in the revised manuscript. (Line # 277 – 284 & 411 – 414).  Concise Data are provided in the revised manuscript  During our study periods all the patients were Negative for HIV tests. Besides, asymptomatic VL patients were not included. |
| 26-    In the discussion section, is not relevant to cite all these paragraphes :  For the clinical aspects, the high prevalence of palpable spleen and severe cytopenia among true VL cases with rk39 positive tests are the most significant aspects that may increase clinical suspicion of VL. The recombinant antigen, rK39 is specific for antibodies against VL caused by L. donovani complex members. It is highly sensitive and predictive for the onset of acute disease [26]. Although the sensitivity of the test is high, it is entirely influenced by the antigen used in the test [10]. In contrast, rK39 does not show detectable antibodies in cutaneous or mucocutaneous leishmaniasis [27]. In this data, all patients had palpable spleen range from 3 to 24cm with a median size of 12cm and 100% of the participants showed rk39 rapid test positive result. | Removed in the revised manuscript |
| 27-    As the actual study used just active VL cases, it’s also not relevant to compare with the other Ethiopian study when the asymptomatic cases were investigated “This is in agreement with a study in south-western Ethiopia, serum cytokines (IFN-γ, IL-12, and IL-15) in symptomatic VL patients were significantly higher than in patients with asymptomatdeeeeeeeeeeic Leishmania infection and healthy controls with significant decrease of IFN-γ and all mediators observed after treatment [12,19]. » | Corrected in the revised manuscript |
| 28-    Please rethink the added value of this study in comparison of what is already known. Such “base-line serum concentration IFN-γ was higher among patients diagnosed with lower parasite load cf. those with higher parasite load” and the cytokines results. is there any difference between man and woman, age groups less of 18 yo and older, HIV test result, the package including the whole definition of a confirmed case of VL compared to the ones who have less symptoms and laboratory threshold levels? And the duration of illness before being confirmed as VL or being visiting conventional health structures. | We believe the analysis of data concerning plasma cytokine concentrations and serum levels of IgG/IgM in relation of parasite grade is new and has yielded interesting results. This observation will be pursued in future studies.  Comparison of antibody levels and cytokine concentrations with respect to such parameters as gender, age groups, duration of illness, symptom severity, and laboratory thresholds, indeed are important. The duration of illness showed a narrow range of variation, 2-4 months, and did not show remarkable association with antibody/cytokine levels (Data not shown). A similar observation is noted when data is analyzed by age groups and gender (S4 Table). We refrained to analyze data concerning severity and laboratory thresholds as there are no guidelines concerning thresholds and severity. All patients were sick, and had abnormal/pathological clinical and laboratory values during admission. |
| 29-    There is no section about the limits of the study. Moreover, some sentences in the conclusion part could be moved to such future limits of the study section | Strength and limitation of the study added in the revised manuscript. |
