# Supplementary material for: Antibody and cytokine levels in visceral leishmaniasis patients with varied parasitemia before, during, and after treatment in patients admitted to Arba Minch General Hospital, southern Ethiopia
Source: PLoS Negl Trop Dis. 2021 Aug 5;15(8):e0009632. doi: 10.1371/journal.pntd.0009632 (PMC8370634; doi:10.1371/journal.pntd.0009632)
Supplement: Attachment — Submitted filename: Responses to the Reviewers comments.docx [file pntd.0009632.s014.docx]

| **Responses to the Reviewers' comments**  First of all, we are very much thankful for their effort to provide small editorial modifications of reviewers which had substantially improved the quality of the manuscript. Reviewers’ suggestions and comments were considered carefully for revising the manuscript. | |
| --- | --- |
| **Editors comment** | **Responses to the Reviewers' comments** |
| **Reviewer #2:** |  |
| Ln 45 The disease is difficult to diagnose >>>> is better to use the condition rather than the disease | Rephrased and revised in the revised manuscript. |
| Ln 46-47 We investigated how the immune response generated during different stages of active VL before, during, and post-treatment was influenced by the presence of different cytokines.  >>>>> different is repeated twice in the same sentence. | Rephrased and revised in the revised manuscript. |
| Ln 83-85 disease and progression [15]. The production of TGF-β by infected macrophages is associated with inhibition of IFN-γ production, suppression of macrophage activation, and progression of disease processes. >>> progression could be replaced by advancement | Rephrased in the revised manuscript |
| Ln 172 to correct plate to plate & day to day variations. >>>> May suggest a short sentence like : to correct variations. | Corrected |
| Ln 368-369 while IFN-γ levels were sustained for longer periods before it started to decline substantially >>> decrease rather than deciline | Corrected |
| Ln 381  at which stage the declines were statistically significant  >>>> reductions rather than declines | Corrected |
| Ln 399-400 these elevations during initial diagnosis/treatment and after treatment were also high. >>>> increased rather than high | Corrected |
| Ln 459 S1 Fig: Spleen size of VL patients at day 0 and end of treatment with varied grades of parasitemia at day 0.>>> various rather than varied | Corrected |
| Ln 115-118 The study population comprised of all VL cases who would be diagnosed based on composite diagnostic criteria [CoDC] (i.e., clinical or serological diagnosis combined with favorable therapeutic response or parasitology by smear and/or culture).  >>>> .. comprised all VL cases .......... reformulate for more clarety the sentence "combined with favorable therapeutic response or parasitology by smear and/or culture" | Reformulated in the revised manuscript |
| Ln 119 the definitive diagnosis was by either of the following >>>> please reformulate | Reformulated in the revised manuscript |
| Ln 135 Prior to studyenrollment >>>> Before the study enrollement | Corrected |
| Ln 139 -141 Following preliminary positive screening test by rk39, clinical and haematological investigations as well as direct microscopic examination of splenic or bone marrow aspiration  were done systematically. >>>> Following a preliminary positive screening test by rk39, clinical and haematological investigations and direct microscopic examination of splenic or bone marrow aspiration  were done systematically. | Corrected |
| Ln 145 Finding of Leishman Donovan bodies in in tissue aspirates >>>> reformulate please | Reformulated in the revised Manuscript |
| Ln 147 were also captured, which include testing for HIV  >>>> ... including testing ... | Corrected |
| Ln 148 -150 After obtaining informed consent, 7.5 ml of whole blood was also collected by venepuncture in a plain and sodium-EDTA vacutainer (Becton, Dickinson, UK) from each study subject. >>>> Please reformulate the sentence for more clarety and easy understanding | Reformulated in the revised Manuscript |
| Ln 150 from each study subject.  >>>> the participant or the control blood subject? to clarify please within the sentence if possible. | Corrected |
| Ln 155 Bone marrow or splenic tissue specimens were collected by experienced physicians. >>>> Experienced physicians collected bone marrow or splenic tissue specimens. | Corrected |
| Ln 159-160 Amastigotes were first counted by two microscopists, and averages obtained. >>>> Two microscopists first counted amastigotes, and averages obtained. | Corrected |
| Ln 167 The ELISA method is based on the detection of antibodies in  serum samples >>>> .....on detecting antibodies ... | Corrected |
| Ln 183 to enable estimation of cytokine >>> to estimate cytokines | Corrected |
| Ln 204 The level of significance >>> The significance level | Corrected |
| Ln 213 Examination of patients’ and specimen collections were performed as per the national guidelines and procedures of Ethiopia.  >>>> .... were performed as recommended by the national guidelines and procedures. | Corrected |
| Ln 346-347 It was implicated that cytokines may be used as biomarkers to monitor the clinical progression of VL and treatment follow up >>>> . It was implicated that cytokines may be used as biomarkers to monitor VL and treatment follow-up's clinical progression | Corrected |
| Ln 357 It is thus >>>>???? | Reformulated in the revised manuscript |
| 1- In the section (inserted at the end of the manuscript , "Strengths and Limitations:  Even though this is a well-designed study that examined the relationships between clinical presentation, parasitaemia, immune progression, response to treatment and parasite clearance, it was limited in the array of mediators selected. Investigations of other mediators, e.g. IL-4, IL-5, IL-6, IL-12, IL-18, TNF-α and others have not been included. Future studies will help to identify additional immune mediators of pathogenesis and biomarkers of prognoses."  >>> the authors are invited to take only the main message and insert it before the conclusion section. | Strengths and Limitations part is inserted into the conclusion part in the revised manuscript |
| 2- in the study participants and sampling section:  A total of 48 patients admitted in LRTC for VL treatment who fulfilled the inclusion criteria were enrolled consecutively. Patients were treated either for 17 days if they received a combination treatment of Sodium Stibogluconate (SSG) and Paromomycin (PM) or for 30 days if they received SSG monotherapy. Patients were followed till end of treatment (EoT)  and samples were collected before treatment at day 0, during treatment at days 7 & 14, at EoT (day 18 or 30), and 120 days after EoT.  The Ethiopian treatment guideline recommends a 17-day combination treatment of SSG (20mg/kg b.wt) and PM (15mg/kg b.wt). During this study period 11 patients received a 30-day dose of SSG as paromomycin was not available for all patients.  >>>> please reformulate the treatment administred like for example:  A total of 48 patients admitted in LRTC for VL treatment who fulfilled the inclusion criteria were enrolled consecutively. the mean treatment provided was the one recommanded by the Ethiopian MoH guideline combining (20mg/kg b.wt) of Sodium Stibogluconate (SSG) and (15mg/kg b.wt) of Paromomycin (PM) for 17 days. However, due to shortcut of PM during the study period 11 patients from the 48 ones received only SGG for the same dose with an extention of duration until 30 days.  >>>> Please add the meaning of "b.wt" in the abbreviation section | Sentences are reformulated and abbreviations are added in the revised manuscript. |
| 3- Ln 131 For analyses of cytokines and antibodies, plasma of 20 blood donors was procured from the Ethiopian National Blood Bank.  >>> PLEASE add : For control values of cytokines and antibodies analyses, plasma of 20 healthy blood donors was procured .... | Reformulated in the revised manuscript |
| 4- In the method section from Ln 152  Serum/plasma samples were stored in a –20°C freezer when they were to be examined within 3-10 months; and/or in a –80°C deep freezer when they were to be analyzed after a longer period (i.e., >10 months).  >>>> Is the ethical commitee agreed of this storage conditions ? | Further analysis and study consent were taken from all study participants and explanations added in the ethical part. |
| 5- Moreover, english editing of the whole manuscript seems important to be done. | Appropriate grammatical and editorial correction is made in the revised manuscript |
